# Cultivar Differences and Impact of Plant-Plant Competition on Temporal Patterns of Nitrogen and Biomass Accumulation

**DOI:** 10.3389/fpls.2019.00215

**Published:** 2019-02-25

**Authors:** Emily Jane Schofield, Jennifer K. Rowntree, Eric Paterson, Mark J. Brewer, Elizabeth A. C. Price, Francis Q. Brearley, Rob W. Brooker

**Affiliations:** ^1^The James Hutton Institute, Aberdeen, United Kingdom; ^2^School of Science and the Environment, Manchester Metropolitan University, Manchester, United Kingdom; ^3^Biomathematics and Statistics Scotland, Aberdeen, United Kingdom

**Keywords:** *Hordeum* sp. (Barley), nitrogen, nutrient uptake, peak accumulation rate, plant-plant competition, plant community coexistence, temporal dynamism

## Abstract

Current niche models cannot explain multi-species plant coexistence in complex ecosystems. One overlooked explanatory factor is within-growing season temporal dynamism of resource capture by plants. However, the timing and rate of resource capture are themselves likely to be mediated by plant-plant competition. This study used Barley (*Hordeum* sp.) as a model species to examine the impacts of intra-specific competition, specifically inter- and intra-cultivar competition on the temporal dynamics of resource capture. Nitrogen and biomass accumulation of an early and late cultivar grown in isolation, inter- or intra- cultivar competition were investigated using sequential harvests. We did not find changes in the temporal dynamics of biomass accumulation in response to competition. However, peak nitrogen accumulation rate was significantly delayed for the late cultivar by 14.5 days and advanced in the early cultivar by 0.5 days when in intra-cultivar competition; there were no significant changes when in inter-cultivar competition. This may suggest a form of kin recognition as the target plants appeared to identify their neighbors and only responded temporally to intra-cultivar competition. The Relative Intensity Index found competition occurred in both the intra- and inter- cultivar mixtures, but a positive Land Equivalence Ratio value indicated complementarity in the inter-cultivar mixtures compared to intra-cultivar mixtures. The reason for this is unclear but may be due to the timing of the final harvest and may not be representative of the relationship between the competing plants. This study demonstrates neighbor-identity-specific changes in temporal dynamism in nutrient uptake. This contributes to our fundamental understanding of plant nutrient dynamics and plant-plant competition whilst having relevance to sustainable agriculture. Improved understanding of within-growing season temporal dynamism would also improve our understanding of coexistence in complex plant communities.

## Introduction

Niche differentiation is suggested to lead to coexistence of plants by reducing competition, either for a specific form of a resource or simultaneous demand for the same resource (Silvertown, 2004). However, in complex plant communities such as rain forests and grasslands there are seemingly insufficient niches to explain coexistence of the many species present. Plants seem to occupy the same niche dimensions but without it leading to competitive exclusion (Clark, 2010).

One factor which is often not included in niche models is time, more specifically the temporal dynamism of key developmental and physiological processes such as resource capture (Schofield et al., 2018). Competition can be influenced by temporally dynamic physiological processes (Poorter et al., 2013), such as flowering (Kipling and Warren, 2014) and nutrient uptake (Jaeger et al., 1999). Differences in the temporal dynamics of nutrient capture could reduce temporal niche overlap, reducing competition for resources. This could result in increased complementarity and promote coexistence (Ashton et al., 2010).

As well as temporal dynamism influencing competition, competition can influence the temporal dynamics of resource capture, although the extent to which these processes affect each other is unclear. As there are many aspects of temporal dynamism in plant communities that are not fully understood, temporal dynamism in resource capture may be currently unsuitable as an indicator of plant-plant competition. However, a change in the temporal dynamics of resource capture may be a wider consequence of competition or a mechanism by which plants avoid direct competition for resources. Trinder et al. (2012) found a change in the temporal dynamics of nitrogen and biomass accumulation in response to inter-specific plant-plant competition. But the impact of competition on temporal dynamism in resource capture, and how this could influence coexistence in plant communities, remains largely unexplored (Schofield et al., 2018).

There is in particular a lack of information on the relationship between temporal dynamism and intra-specific competition, and how the degree of relatedness of competitors might influence temporal dynamism. The genetic distance between competing individuals can influence the functional plasticity of an individual response to competition (Murphy et al., 2017), including biomass allocation and root morphology (Semchenko et al., 2017). Differential competitive responses have been demonstrated between closely related individuals (Murphy et al., 2017), including in a number of crop species (Dudley and File, 2007). The use of two cultivars in this study allows a tight control of the relatedness of individuals, which in turn allows us to address how diversity regulates interactions and ultimately functions in a range of systems [not least for the development of sustainable agricultural practice (Schöb et al., 2018)]. In this sense, crop species are ideal model systems for undertaking such studies.

Here, we conducted a pot experiment with Barley (*Hordeum vulgare*) as a model species, using an early and a late cultivar. Barley is a suitable model in this case as its nutrient uptake has been studied in detail to optimize the timing of fertilizer application in agriculture (Nielsen and Jensen, 1986), allowing us to address fundamental ecological questions of plant coexistence, as well as investigating a topic of relevance for agricultural practices.

It is expected that early and late cultivars of barley will have different temporal dynamics of nitrogen uptake and biomass accumulation, in a similar way to two species or genotypes in a natural system. The two cultivars in this study have been bred for different uses and therefore will have differing combinations of traits. Tammi has been bred for an early lifecycle (Nitcher et al., 2013), whereas Proctor was bred for malting quality (Hornsey, 2003). The nitrogen uptake and biomass accumulation dynamics are predicted to be altered by plant-plant competition, and this will be more pronounced in intra-cultivar compared to inter-cultivar competition as the individuals will more completely occupy the same niche space.

This study aimed to understand: (1) whether early and late cultivars of barley exhibit temporal dynamics in nitrogen uptake and biomass, (2) how plant-plant competition changes the temporal dynamics of nitrogen and biomass accumulation in early and late barley cultivars, (3) how any temporally dynamic response differs with inter- and intra- cultivar competition, and ultimately (4) how this impacts on niche complementarity.

## Materials and Methods

### Temporal Patterns of Nitrogen and Biomass Accumulation

A pot-based competition study was used to investigate temporal dynamism in nitrogen uptake, using barley (*Hordeum* sp.) as a model species. An early (Tammi: T) and late (Proctor: P) cultivar of barley (sourced from The James Hutton Institute, Dundee, United Kingdom) were chosen as they have similar height and limited tillering, enabling the study to focus on phenological rather than physiological differences. Each cultivar was grown in pots either in isolation, or with another individual of either the same or other cultivar (i.e., T, P, TT, PP, and TP).

### Soil Characteristics

Soil was sourced from an agricultural field (Balruddery Farm, Invergowrie, United Kingdom) that had previously contained spring barley (*Hordeum* sp.) and had been subject to standard management for barley production (including fertilizer addition at a rate of 500 kg of 22N-4P-14 K ha^-1^ year^-1^). The soil had an organic matter content of 6.2 ± 0.3% SEM (*n* = 4), a mean pH (in water) of 5.5 ± 0.02 SEM (*n* = 4), a total organic nitrogen concentration of 0.078 ± 0.024 mg l^-1^, mean NH_4_ concentration of 0.008 ± 0.006 mg l^-1^ and mean NO_3_ concentration of 0.078 ± 0.024 mg l^-1^ (*n* = 4) and microbial biomass of 0.06 ± 0.002 SEM mg g^-1^ (*n* = 4) [analyzed by Konelab Aqua 20 Discrete Analyser (Thermo Fisher Scientific, Waltham, MA, United States)]. Before use, the soil was passed through a 6 mm sieve. No fertilization of the soil occurred during the experiment.

### Setup and Growing Conditions

Seeds of both cultivars were pre-germinated in the dark on damp paper towels and planted into cylindrical 2 L pots (diameter 152 mm, height 135 mm) with five replicate pots of each of the five treatments for each planned harvest (11 harvests in total), giving a total of 275 pots. The pots were randomized to account for potential positional effects and grown in controlled environment rooms (Conviron, Isleham, United Kingdom) at a constant 15°C with an 8/16 (day/night) hour photoperiod (irradiance of 100–150 μmol m^-2^ s^-1^) and 65% relative humidity, to mimic local spring-time conditions. The pots were watered twice weekly and the soil was kept moist to avoid competition for water. Mesh screens [45 cm × 16 cm, mesh size 0.08 mm (Harrod Horticulture, Lowestoft, United Kingdom)] were inserted in those pots containing two plants to separate the plants above ground, and ensure competitive interactions only occurred below ground. Foliage was relatively upright without support and the presence of a screen – although important in ensuring above-ground competition was minimized – was unlikely to have resulted in differences in shoot development in pots with two plants compared to one.

### Sequential Harvesting

Five randomly selected pots of each treatment were harvested every 5 days until ear formation (when grain begins to form) was observed on the early Tammi cultivar (60 days). During this period both cultivars produced flag leaves, the stage prior to grain production, when most nitrogen has already been absorbed (Spink et al., 2015). This covered the period most likely to contain the peak nitrogen and biomass accumulation rate for both cultivars, the focus of this study. The plants were then removed from the pots, the roots washed, and individual shoot and root material separated. The root and shoot material of each plant were dried at 30°C until a stable weight was reached and weighed. Milled shoot samples were analyzed for carbon and nitrogen concentration (Flash EA 1112 Series, Thermo Fisher Scientific, Bremen, Germany).

### Data Analysis

#### Temporal Patterns of Nitrogen and Biomass Accumulation

To analyze temporal changes in biomass and nitrogen accumulation, the rate of each was modeled with logistic growth curves using non-linear least squares (nls) models (R Core Team, 2015). A cumulative time series data set of biomass accumulation was bootstrapped using resampling with replacement 1000 times to estimate variability and confidence intervals. A logistic growth curve was used as the nls model and this was fitted to each of the bootstrapped data sets to produce a set of logistic instantaneous uptake rate curves for each treatment, as well as sets of modeled maximum accumulation values. This was then repeated for the nitrogen accumulation data set. A non-linear model was used as the growth dynamics of plants with determinate growth such as barley (Yin et al., 2003) are mostly sigmoidal, making a linear growth model unsuitable (Robinson et al., 2010). Therefore, the use of the non-linear least squares model with bootstrapping is a robust method to examine the temporal dynamism of resource capture of annual species and to properly account for uncertainty. Significant differences between the timing of peak accumulation and final maximum accumulation between treatments were determined from the difference in bootstrapped 95% confidence intervals of the model outputs (Supplementary R Code 1).

##### Shoot C:N

C:N ratio at the final harvest (65 days after planting) was analyzed using an ANOVA test from the MASS package *in R* (R Statistical Software, R Core Team, 2015) as the residuals were normally distributed, with treatment as the fixed factor and C:N as the response variable (Supplementary R Code 2). A Tukey *post hoc* test was carried out to compare the individual treatment groups.

#### Neighbor Effects

The effect of a neighboring plant on a target plant’s biomass was quantified using the Relative Intensity Index (RII; Eq. 1), an index that accounts for both competitive and facilitative interactions between neighboring plants (Díaz-Sierra et al., 2017). RII was calculated using the final harvest biomass data. For each cultivar, RII was calculated separately for plants grown in intra- and inter-specific competition. The mean total biomass of each cultivar grown in isolation was used for the *Isolation* value, and the individual RII value was then calculated for each plant of that cultivar experiencing competition.

(1) $$RII=\frac{\left(Competition-Isolation\right)}{\left(Competition+Isolation\right)}$$

*Competition* = Biomass of plant when in competition*, Isolation* = Mean biomass of plant in isolation.

The land equivalent ratio (LER; Eq. 2) was used to determine if the inter-cultivar mixture (TP) overyielded when compared to intra-cultivar competition (TT or PP) (Mead and Willey, 1980). The mean LER value was calculated by randomly pairing inter- and intra- cultivar competition treatments using a random number generator. A LER value was calculated for each pairing, from which a mean and SEM was calculated. A mean LER value above 1 indicates that inter-cultivar pairings produced more biomass than to intra-cultivar combinations. As the residuals were normally distributed, the LER and RII values were compared between competition treatments using an ANOVA test as above, with treatment as the fixed factor and either LER or RII as the response variable (Supplementary R Code 2).

(2) $$LER=\frac{Tammi\;mixture\;biomass}{Tammi\;own\;cultivar\;biomass}+\;\frac{Proctor\;mixture\;biomass}{Proctor\;own\;cultivar\;biomass}$$

*Tammi mixture biomass* = Tammi biomass when in competition with Proctor, *Tammi own cultivar biomass* = Tammi biomass of the focal plant when in competition with another Tammi. *Proctor mixture biomass* = Proctor yield when in competition with Tammi, *Proctor own cultivar biomass* = Proctor biomass when in competition with another Proctor.

## Results

**FIGURE 1:**
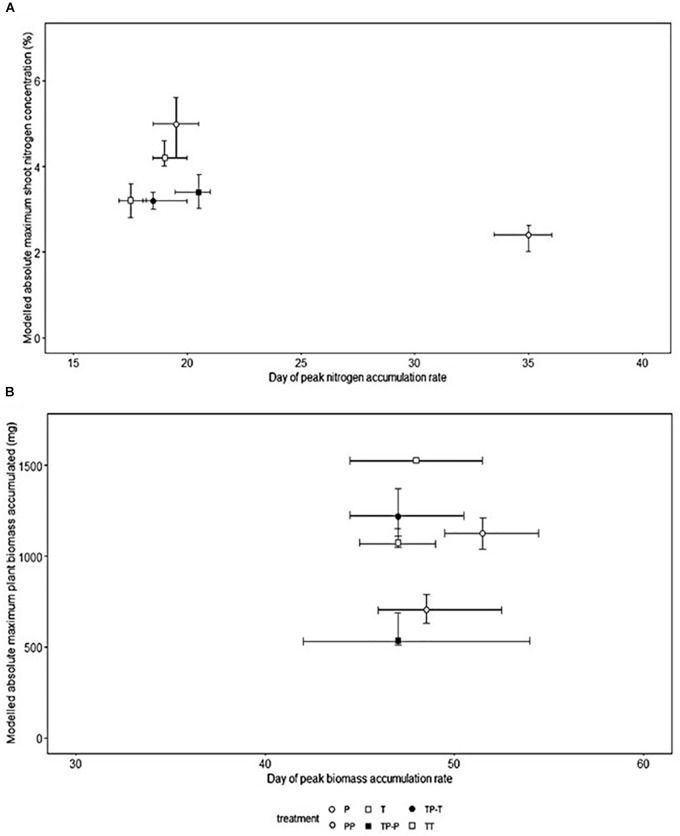
Timing of peak nitrogen **(A)** and biomass **(B)** accumulation rate, the shoot nitrogen concentration and absolute maximum accumulated total biomass at the end of the experiment in barley (*Hordeum* sp.). Bootstrapped modeled accumulation derived from non-linear least squares model (T, Tammi; P, Proctor; TP-T, Tammi in competition with Proctor; TP-P, Proctor in competition with Tammi; TT, Tammi own cultivar competition; PP, Proctor own cultivar competition). Error bars represent the 95% confidence intervals derived from the non-linear least squares model.

Nitrogen (Figure 1A) and biomass (Figure 1B) accumulation were temporally distinct for both cultivars. The peak rate of nitrogen accumulation occurred between 17.5 and 19.0 days after planting for Tammi and 19.5–35.0 days for Proctor. The peak rate of biomass accumulation occurred between 47 and 48 days after planting for Tammi and 47.0–51.5 days for Proctor (Model details in Supplementary Table 1).

### Temporal Dynamics of Nitrogen Uptake

**FIGURE 2:**
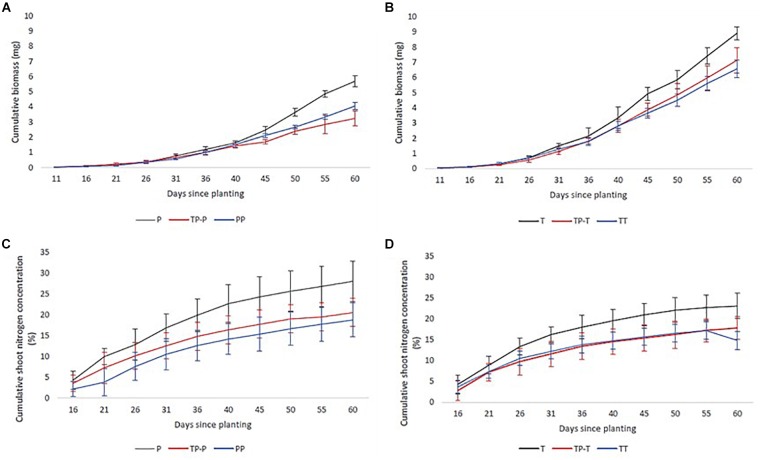
Mean cumulative nitrogen **(C,D)** and biomass **(A,B)** accumulation of Tammi and Proctor barley cultivars over time. Pots contained Proctor in isolation (P), in competition with Tammi (TP) and in competition with another Proctor (PP), Tammi in isolation (T), in competition with Proctor (TP) and another Tammi (TT). Error bars are two times the SEM.

Nitrogen uptake for both cultivars followed similar temporal dynamics, increasing until 45 days after planting, then plateauing (Figure 2A,B). There was no significant change in the timing of peak nitrogen uptake rate in response to inter-cultivar competition for either cultivar. However, both cultivars showed a significant shift in peak accumulation rate in response to intra-cultivar competition (Figure 1A). Tammi demonstrated an advance in peak uptake rate by 0.5 days and Proctor a delay of 14.5 days (Supplementary Table 2).

#### Maximum Accumulated Shoot Nitrogen

Proctor’s absolute maximum shoot nitrogen concentration was significantly lower when in competition with Tammi or Proctor compared to isolation (Figure 1A). Inter-cultivar competition caused a significantly lower maximum shoot nitrogen concentration compared to intra-cultivar competition for Proctor but not Tammi. Intra-cultivar competition caused a significantly lower maximum shoot nitrogen concentration for Tammi but not Proctor (Supplementary Table 3).

### Temporal Dynamics of Biomass Accumulation

Biomass accumulation increased throughout the growing period with a lag period until 31 days after planting and then rapidly increased during the remainder of the experiment (Figure 2C,D). In response to competition, Tammi did not exhibit a shift in peak biomass accumulation rate, with peak accumulation rate always occurring 47–48 days after planting. Proctor biomass accumulation rate peaked between 48 and 51.5 days after planting (Figure 1B); although there was a trend toward an earlier peak in biomass accumulation when in competition there were no significant differences between treatments (Supplementary Table 2).

#### Maximum Accumulated Total Plant Biomass

For both Tammi and Proctor, absolute maximum accumulated biomass was significantly lower when in competition compared to isolation (Figure 1B). However, neither cultivar demonstrated a significant difference between intra- and inter- cultivar competition in maximum accumulated biomass (Supplementary Table 3).

#### Shoot C:N

Proctor in isolation had a C:N ratio of about half that of Tammi in isolation throughout the experiment, i.e., more nitrogen relative to carbon. However, for neither cultivar were there significant differences in C:N ratio between plants in isolation compared to plants in competition at the end of the experiment [Proctor (*F*_(2,17)_ = 1.44, *P* = 0.26); Tammi (*F*_(2,17)_ = 2.74, *P* = 0.09)] (details in Supplementary Table 4).

### Neighbor Effects

**FIGURE 3:**
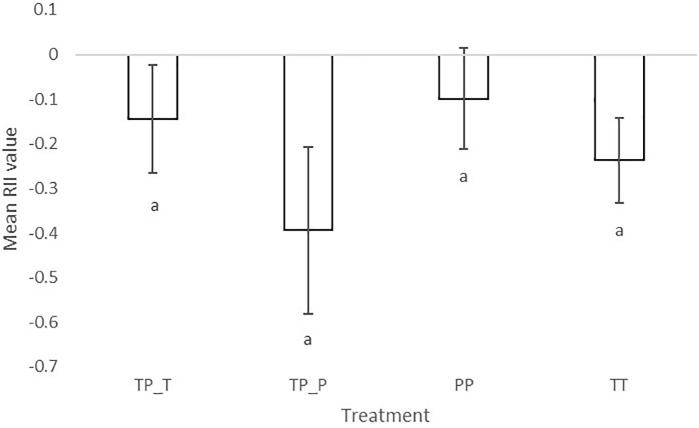
Mean relative intensity index of barley (*Hordeum* sp.) Tammi and Proctor cultivars in inter- and intra-cultivar competition. The more negative the result the greater competition the plant experienced. TP-T, Tammi in inter-cultivar competition; TP-P, Proctor in inter-cultivar competition; TT, Tammi in intra-cultivar competition; PP, Proctor in intra-cultivar competition. Error bars are two times the SEM. Letters indicate significant differences from a Tukey *post hoc* test.

The significantly negative RII of final biomass indicated competitive interactions for both cultivars irrespective of whether they were in inter- or intra- cultivar mixtures. RII values also showed that Tammi and Proctor experienced a greater intensity of competition when in inter-cultivar compared to intra-cultivar competition (Figure 3). Proctor in intra-cultivar competition experienced the lowest intensity of competition; however, there was no significant difference between the competition treatments [*F*_(3,26)_ = 2.86, *P* = 0.06].

The LER value for Tammi and Proctor in competition was 2.05 (±0.35 SE), indicating that the inter-cultivar mixture had a greater total biomass (root and shoot) than would be expected from the intra-cultivar mixtures.

## Discussion

This experiment aimed to detect and quantify temporal dynamism in nitrogen uptake and biomass accumulation in two barley cultivars and determine responses to inter- and intra- cultivar competition.

We found that competition significantly reduced maximum accumulated biomass and shoot nitrogen in both cultivars. Neither intra- or inter-cultivar competition impacted the timing of peak biomass accumulation in either cultivar. However, intra-cultivar competition significantly delayed peak nitrogen accumulation rate by 14.5 days in Proctor and advanced it in Tammi by 0.5 days. Relative Intensity Index values indicated that both cultivars experienced competition, with no significant difference in intensity between intra- and inter- cultivar competition. However, a positive LER value indicated that the inter-cultivar mixture overyielded when compared to the intra-cultivar mixtures.

### Shifts in the Timing of Biomass Accumulation in Response to Competition

Neither of the cultivars in this study significantly altered the temporal dynamics of peak biomass accumulation in response to a competitor. The mismatch between biomass and nitrogen accumulation dynamics in response to competition indicates biomass may not effectively measure the temporal dynamics of within-growing season resource capture, an issue previously raised by Trinder et al. (2012).

### Shifts in the Timing of Nitrogen Accumulation in Response to Competition

Tammi and Proctor only demonstrated significant changes in temporal dynamism of nitrogen accumulation when in intra-cultivar competition. Tammi advanced peak accumulation rate by 0.5 days and Proctor delayed it by 14.5 days. As this only occurred in intra-cultivar competition, it suggests that this is more complex than a competition avoidance response based on a source-sink (soil – plant) relationship. If this was a simple source-sink relationship, for example, based on soil nitrogen availability (Dordas, 2009), the inter- and intra-cultivar responses to competition should be identical. However, a response to only intra-cultivar competition suggests a kin recognition mechanism.

Kin recognition has been suggested as a mechanism by which plants alter functional traits when in competition with closely related individuals (Sousa-Nunes and Somers, 2010). It has been found to most commonly be mediated belowground through root exudates (Biedrzycki et al., 2010; Bais, 2015). This may mediate specific responses depending on the identity of a competing plant, as found in this study.

The results of this study contrast with those of a temporal dynamism study by Trinder et al. (2012) which examined the influence of interspecific competition on the temporal dynamics of nitrogen uptake and biomass accumulation using *Dactylis glomerata* and *Plantago lanceolata*, two perennial grassland species. *D. glomerata* was the later of the two species, and *P. lanceolata* the earlier species. They found a 7 days delay for *D. glomerata* and a 5 days advancement for *P. lanceolata* in maximum biomass accumulation rate in competition compared to plants in isolation, with a similar pattern of divergence for peak nitrogen accumulation rate. We did not find these trends between two cultivars, with no significant shifts in peak biomass accumulation rate and a significant delay in peak nitrogen accumulation rate only when Proctor was in own cultivar competition.

In our study Proctor was the less competitive of the two cultivars, as it experienced a greater decrease in nitrogen and biomass accumulation when in competition compared to Tammi. This contrasts with the Trinder et al. (2012) study which found that *D. glomerata* took up the most nitrogen and it could be argued was therefore the most competitive, despite being the later species for peak nitrogen and biomass accumulation rate. Therefore, it should not be assumed that the earlier species or cultivar is automatically the most competitive.

Trinder et al. (2012) also found that competition reduced the period between peak nitrogen and biomass accumulation rate compared to plants in isolation, from 10 to 1 days for *D. glomerata*, and from fourteen to 3 days for *P. lanceolata*. We also found this effect, but only when Proctor was in competition, which caused a shortening of the period between peak rate of nitrogen uptake and biomass accumulation by 18.5 days in intra-cultivar competition and 5.5 days when in inter-cultivar competition. However, the reason for this response is unclear. It could be a phenological change in response to competition, a pattern previously observed in cases of abiotic stress (Kazan and Lyons, 2016) and pathogen attack (Korves and Bergelson, 2003).

#### Temporal Segregation of Nitrogen and Biomass Accumulation

The processes of nitrogen and biomass accumulation were temporally distinct for both cultivars. The peak rate of nitrogen accumulation was 29.0–29.5 days before peak biomass accumulation for Tammi and 16.5–27.5 days for Proctor (Figure 1). The gap between peak nitrogen and biomass accumulation was less variable for Tammi compared to Proctor. Tammi was specifically bred for an early phenotype (Nitcher et al., 2013), whereas Proctor was bred for malting quality (Hornsey, 2003). This selection pressure for phenology in Tammi may go some way to explaining the lack of variability in the gap between peak nitrogen and biomass accumulation in response to competition. Future studies could investigate whether similar response patterns are found in the genotypes of wild species or in wild species with contrasting phenologies.

Barley has been found to have temporally distinct nitrogen and biomass accumulation with a 23–24 day gap between peak nitrogen and biomass accumulation in field studies (Malhi et al., 2006). The gap between the peak nitrogen and biomass accumulation rate was shortened when Proctor was in competition, indicating the impact of plant-plant competition on the temporal dynamics of nitrogen accumulation. The greatest reduction in the gap between peak nitrogen and biomass accumulation rate occurred when Proctor was in intra-cultivar competition. This was also the treatment with the lowest absolute shoot nitrogen concentration, suggesting delaying peak rate of nitrogen accumulation for this cultivar is a response to intra-cultivar competition.

### Impact of Competition on Final Nitrogen and Biomass Accumulation

Competition significantly reduced the final maximum nitrogen concentration and biomass that both Proctor and Tammi were able to accumulate in intra- or inter-cultivar competition. A Proctor competitor caused a significant decrease in Tammi maximum biomass accumulation and nitrogen shoot concentration, despite not achieving the greatest biomass above or below ground. This suggests that another factor influenced the rate of nitrogen uptake. Signaling through root volatile compounds or root exudates has been found in a number of species including legumes and grasses (Pierik et al., 2013) and may be acting here. Plant root exudates select for a specific microbial community (Shi et al., 2016) and have been found to affect the rate of microbial soil organic matter turnover (Mergel et al., 1998). Therefore, plants may influence the timing of soil microbial community activity in order to reduce direct competition for resources. However, as we are only starting to understand the role of short term-temporal dynamism in plant interactions (Schofield et al., 2018) it is not surprising that further studies are required to determine the role of the root exudates in neighbor recognition and temporally dynamic responses, and why this response is greater for intra- compared to inter- specific competition.

### Shoot C:N in Response to Identity of a Competing Individual

The two cultivars differed in their C:N ratio by the end of the experiment. This is likely due to the earlier cultivar Tammi being more advanced developmentally than Proctor. By the end of the experiment, Tammi had begun grain production, whereas Proctor had produced a flag leaf, the stage before grain formation. However, there was no significant increase in C:N in either cultivar in response to competition. Due to selective breeding for a specific seed C:N (grain nitrogen content) with known mapped genes (Cai et al., 2013) it is unlikely that C:N is highly plastic in barley, making it a poor measure of competition in this case.

### Is Greater Complementarity Achieved?

The negative RII indicated both cultivars experienced competition when grown with a neighboring plant, but no significant difference depending on the identity of the competitor. This contrasts with the positive LER value which indicated overyielding of the two cultivars when grown in inter-cultivar competition compared to intra-cultivar competition. The reason for this is unclear and may be due to the timing of the final harvest, before both cultivars had set seed. This highlights the difficulty of using multiple metrics to measure the outcome of competition, especially as the measurements were only taken at the end of the experiment, i.e., at a single timepoint. Therefore, single timepoint competition indices should be used with caution when examining the consequences of temporal dynamism of resource capture.

There is a need to understand the extent to which a species or genotype is temporally dynamic and the factors that lead to temporal dynamism in resource capture. This will allow temporal dynamism in resource capture to be included in models of coexistence, furthering our understanding of coexistence in complex plant communities.

## Conclusion

This study demonstrates how a previously overlooked factor in plant community coexistence, within-growing season temporal dynamism of resource capture, can be measured through successive harvesting and the novel application of commonly used statistical approaches. Only peak nitrogen accumulation rate was temporally dynamic in response to competition, not biomass peak accumulation rate or shoot C:N. Therefore, we suggest that to understand the temporal dynamics of resource capture within a growing season, direct measures of mineral resources accumulated (e.g., nitrogen uptake) are important to understand the mechanisms of temporally dynamic responses to competition. By measuring shoot nitrogen accumulation rate over time, intra-cultivar competition was found to advance peak nitrogen accumulation rate in Tammi and delay it in Proctor. This suggests that temporally dynamic nitrogen uptake responses are greater in intra-cultivar competition and may be due to kin recognition. This may be mediated through root exudates and the soil microbial community, an area that requires further investigation and extension to semi-natural and natural ecosystems. Ultimately understanding the role of temporal dynamism in plant communities will lead to improved niche models of coexistence in plant communities.

## Data Availability

The datasets generated for this study can be found in the Dryad Digital Repository.

## Author Contributions

ES, RB, JR, EP, FQB, and EACP conceived the experimental design. ES collected the data. RB, MB, JR, and ES analyzed the data. All authors wrote and/or edited the manuscript.

## Conflict of Interest Statement

The authors declare that the research was conducted in the absence of any commercial or financial relationships that could be construed as a potential conflict of interest.
