# Supplementary material for: Cultivar Differences and Impact of Plant-Plant Competition on Temporal Patterns of Nitrogen and Biomass Accumulation
Source: Front Plant Sci. 2019 Feb 25;10:215. doi: 10.3389/fpls.2019.00215 (PMC6397874; doi:10.3389/fpls.2019.00215)
Supplement: Supplementary file 1 [file Table_1.DOCX]

Supplementary Material

**Cultivar differences and impact of plant-plant competition on temporal patterns of nitrogen and biomass accumulation**

**E.J. Schofield^1,2^*, J. K. Rowntree ^2^, E. Paterson^1^, M.J. Brewer^3^, E. A. C. Price ^2^, F. Q. Brearley ^2^, R. W. Brooker^1^**

*** Correspondence:** E.J. Schofield: Emily_schofield@hutton.ac.uk

# Supplementary Data

Supporting R Code 1

The code used to run the nls model with bootstrapping model in R (R Core Team, 2015) analyzing the effect of plant-plant competition on the timing of peak nitrogen and biomass accumulation rate.

n.col <- ncol(Tammi)

n.row <- nrow(Tammi)-1 # line one is time data so subtract

Tammi.time <- as.numeric(Tammi[1,]) # extract the times

Tammi.time <- rep(Tammi.time,each=n.row)

Tammi1 <- ts(Tammi[-1,]) #needs to be a time series to bootstrap correctly

#resampling bootstrap

TammiBoot <- list() #creates list to put values in

for(i in 1:1000){

TammiBoot[[i]] <- Tammi1 # copy the original data to a list entry for TammiBoot (so we get the right size object)

for(j in 1:n.col){

# replace each column of TammiTemp with a resample of the n.row data points at that time

TammiBoot[[i]][,j] <- sample(Tammi1[,j], size=n.row, replace=TRUE)

}

}

TammiBoot

#make data cumulative

TammiBootCumul <- list()

TammiBootforAnalysis <- list()

for(i in 1:1000){

TammiBootCumul[[i]] <- (t(apply(TammiBoot[[i]],1,cumsum)))

TammiBootforAnalysis[[i]] <- c(TammiBootCumul[[i]]) # convert to vector form for nls

}

n.models <- 1000

# run the nls on the bootstrap resamples

Tammi.a <- array(NA,dim=c(n.models,4))

Tammi.b <- array(NA,dim=c(n.models,4))

Tammi.c <- array(NA,dim=c(n.models,4))

TammiBootModels <- list()

for(i in 1:n.models){

Tammi.temp <- TammiBootforAnalysis[[i]]

TammiBootModels[[i]] <- nls(Tammi.temp ~ SSlogis(Tammi.time, a, b, c)) # need to be able to loop this for every line of the matrix

Tammi.a[i,] <- summary(TammiBootModels[[i]])$coef["a",]

Tammi.b[i,] <- summary(TammiBootModels[[i]])$coef["b",]

Tammi.c[i,] <- summary(TammiBootModels[[i]])$coef["c",]

}

colnames(Tammi.a) <- colnames(summary(TammiBootModels[[1]])$coef)

colnames(Tammi.b) <- colnames(summary(TammiBootModels[[1]])$coef)

colnames(Tammi.c) <- colnames(summary(TammiBootModels[[1]])$coef)

# Maximum points

# Mean peak time from the bootstrap (x-axis)

mean(Tammi.b[,"Estimate"])

# CI for peak time from the bootstrap (x-axis)

quantile(Tammi.b[,"Estimate"],probs=c(0.025,0.975)) # 95%

# Calculate vector of bootstrapped peak heights of rate per day (y-axis)

Tammi.peaks <- Tammi.a[,"Estimate"]/(4*Tammi.c[,"Estimate"])

# Mean peak rate from the bootstrap (y-axis)

mean(Tammi.peaks)

# CI for peak rate from the bootstrap (y-axis)

quantile(Tammi.peaks,probs=c(0.025,0.975)) # 95%

#significant differences in timing

Tammi.peaks.T <- Tammi.b

Tammi.peaks.TT <- Tammi.b

Tammi.peaks.TP <-Tammi.b

Proctor.peaks.P <- Tammi.b

Proctor.peaks.TP <- Tammi.b

Proctor.peaks.PP <- Tammi.b

Tammi.peaks.T.minus.Tammi.peaks.TP <- Tammi.peaks.T[sample(1000)] - Tammi.peaks.TP[sample(1000)]

quantile(Tammi.peaks.T.minus.Tammi.peaks.TP,probs=c(0.025,0.975))

Tammi.peaks.T.minus.Tammi.peaks.TT <- Tammi.peaks.T[sample(1000)] - Tammi.peaks.TT[sample(1000)]

quantile(Tammi.peaks.T.minus.Tammi.peaks.TT,probs=c(0.025,0.975))

Proctor.peaks.P.minus.Proctor.peaks.PP <- Proctor.peaks.P[sample(1000)] - Proctor.peaks.PP[sample(1000)]

quantile(Proctor.peaks.P.minus.Proctor.peaks.PP,probs=c(0.025,0.975))

Proctor.peaks.P.minus.Proctor.peaks.TP <- Proctor.peaks.P[sample(1000)]- Proctor.peaks.TP[sample(1000)]

quantile(Proctor.peaks.P.minus.Proctor.peaks.TP,prob=c(0.025,0.975))

#Testing for significant accumulation differences in bootstrapped samples

Tammi.acc.T <- Tammi.peaks

Tammi.acc.TT <- Tammi.peaks

Tammi.acc.TP <- Tammi.peaks

Proctor.acc.P <- Tammi.peaks

Proctor.acc.PP <- Tammi.peaks

Proctor.acc.TP <- Tammi.peaks

Tammi.acc.T.minus.Tammi.acc.TT <- Tammi.acc.T[sample(1000)] - Tammi.acc.TT[sample(1000)]

quantile(Tammi.acc.T.minus.Tammi.acc.TT,prob=c(0.025,0.975))

Tammi.acc.T.minus.Tammi.acc.TP <- Tammi.acc.T[sample(1000)] - Tammi.acc.TP[sample(1000)]

quantile(Tammi.acc.T.minus.Tammi.acc.TP,prob=c(0.025,0.975))

Proctor.acc.P.minus.Proctor.acc.PP <- Proctor.acc.P[sample(1000)] - Proctor.acc.PP[sample(1000)]

quantile(Proctor.acc.P.minus.Proctor.acc.PP,prob=c(0.025,0.975))

Proctor.acc.P.minus.Proctor.acc.TP <- Proctor.acc.P[sample(1000)] - Proctor.acc.TP[sample(1000)]

quantile(Proctor.acc.P.minus.Proctor.acc.TP,prob=c(0.025,0.975))

Supporting R Code 2

Code run in R (R Core Team, 2015) to determine the effect of treatment on shoot carbon to nitrogen ratio (C:N) of plants harvested at 65 days after planting with a Tukey post-hoc test.

CN_65days$treatment <- (CN_65days$treatment)

CN_65days$ID <- (CN_65days$ID)

Res <- aov(CN ~ treatment, data = CN_65days)

anova(Res)

TukeyHSD(Res)

# Supplementary Figures and Tables

| Treatment | Peak timing (days since planting) | Absolute maximum (mg) |
| --- | --- | --- |
| Biomass |  |  |
| T | 48.0 (44.5 - 51.5) | 1527.3 (1342.5 - 1707.2) |
| TT | 47.0 (45.0 - 49.0) | 1069.5 (986.6 - 1149.3) |
| TP-T | 47.0 (44.5 - 50.5) | 1221.8 (1068.8 - 1368.6) |
| P | 51.5 (49.5 - 54.5) | 1125.1 (1042.6 - 1207.0) |
| PP | 48.5 (46.0 - 52.5) | 705.0 (630.7 - 785.1) |
| TP-P | 47.0 (42.0 - 54.0) | 530.9 (530.9 - 687.4) |
| Nitrogen |  |  |
| T | 19.0 (18.5 - 20.0) | 210.0 (190.0 - 220.0) |
| TT | 17.5 (17.0 - 18.0) | 160.0 (150.0 - 180.0) |
| TP-T | 18.5 (17.5 20.0) | 160.0 (150.0 - 170.0) |
| P | 19.5 (18.5 - 20.5) | 210.0 (190.0 - 230.0) |
| PP | 35.0 (33.5 - 36.0) | 120.0 (100.0 - 140.0) |
| TP-P | 20.5 (19.5 - 21.0) | 170.0 (150.0 - 190.0) |

**Supplementary Table 1** – Model parameters of the logistic growth curve fitting using a nls model of biomass and nitrogen accumulation of Proctor and Tammi barley varieties grown in isolation, intra- and inter- cultivar competition. Confidence intervals of 2.5% and 97.5% are shown in brackets.

| Treatment | CI differences in timing of peak accumulation rate |
| --- | --- |
| **Biomass** |  |
| T vs. TT | -9.0, 11.0 |
| T vs. TP-T | -10.0, 11.0 |
| P vs. PP- | -9.0, 16.5 |
| P vs. TP-P | -8.0, 13.0 |
| **Nitrogen** |  |
| T vs. TT | 11.5, 14.0* |
| T vs. TP-T | -12.0, 13.0 |
| P vs. PP | -13.0, 12.5 |
| P vs. TP-P | -33.0, -29.5* |

**Supplementary Table 2 -** Difference between bootstrapped confidence intervals of timing of peak biomass and nitrogen accumulation of Proctor (P) and Tammi (T) barley plants grown in isolation (T, P), inter-cultivar competition (TT, PP) and inter-cultivar competition (Tammi: TP-T, Proctor: TP-P). Asterisks indicate significant differences.

| Treatment | CI differences in maximum accumulation |
| --- | --- |
| **Biomass** |  |
| T vs. TT | 268.10, 653.69* |
| T vs. TP-T | 89.81, 547.62* |
| P vs. PP | 312.00, 523.62* |
| P vs. TP-P | 441.20, 728.07* |
| TP-P vs. PP | -8.82, 302.41 |
| TP-T vs. TT | -332.64, 19.17 |
| **Nitrogen** |  |
| T vs. TT | 0.03, 0.07* |
| T vs. TP-T | 0.03, 0.07* |
| P vs. PP | 0.06, 0.11* |
| P vs. TP-P | 0.01, 0.06* |
| TP-P vs. PP | 1.98, 2.02* |
| TP-T vs. TT | -0.02, 0.02 |

**Supplementary Table 3 -** Difference between bootstrapped confidence intervals of absolute maximum biomass and shoot nitrogen accumulation of Proctor (P) and Tammi (T) barley plants grown in isolation (T, P), inter-cultivar competition (TT, PP) and inter-cultivar competition (Tammi: TP-T, Proctor: TP-P). Asterisks indicate significant differences.

| Proctor | | | | | |
| --- | --- | --- | --- | --- | --- |
|  | Degrees of Freedom | Sum of squares | Mean of squares | F value | P value |
| Treatment | 2 | 203.3 | 101.64 | 1.44 | 0.26 |
| Residuals | 17 | 1196.7 | 70.39 |  |  |
|  |  |  |  |  |  |
| Tukey test | P value | Lower confidence interval | Upper confidence interval |  |  |
| PP - P | 0.27 | -4.50 | 19.08 |  |  |
| TP-P - P | 0.36 | -6.11 | 21.11 |  |  |
| TP-P - PP | 0.99 | -11.58 | 0.99 |  |  |
| Tammi | | | | | |
| Treatment | 2 | 2915 | 1457.6 | 2.74 | 0.09 |
| Residuals | 17 | 9053 | 532.5 |  |  |
|  |  |  |  |  |  |
| Tukey test | P value | Lower confidence interval | Upper confidence interval |  |  |
| TP-T - T | 0.08 | -71.41 | 3.47 |  |  |
| TT - T | 0.50 | -46.96 | 17.89 |  |  |
| TT - TP-T | 0.30 | -12.98 | 51.87 |  |  |

**Supplementary Table 4** – Model parameters of the ANOVA and Tukey post-hoc test analysis carried out on shoot C:N of Proctor and Tammi barley varieties grown in isolation, intra- and inter- cultivar competition.
